# Stress adaptation and resilience of academics in higher education

**DOI:** 10.1007/s12564-023-09829-1

**Published:** 2023-02-22

**Authors:** P. M. Ross, E. Scanes, W. Locke

**Affiliations:** 1 University of Melbourne, Parkville, Melbourne, VIC Australia; 2 The University of Sydney of School Life and Environmental Science, Sydney, NSW Australia; 3 University of Technology, Climate Change Cluster, Sydney, NSW Australia

**Keywords:** Higher education, Stress, Resilience, Adaptation, Academics, COVID-19

## Abstract

Academics in higher education around the world indicate high levels of stress from multiple sources. The COVID-19 pandemic has only served to intensify stress levels. Adaptation and resilience are needed if academics, particularly those focused on education and teaching, are to endure, learn, and *bounce back* during this era of stress and contribute to education quality and student learning. This review is organized to answer two key questions. First, what are the main forms of stress for academics, especially those focused on education and teaching? Second, what are the responses of academics to stress and is the concept of resilience relevant to understand the consequences for academic careers oriented toward education and education quality? To answer these questions, we first critically review the literature on the responses of academics to stress and the concept of resilience, which has been employed by multiple disciplines, including teacher education. We then broadly define the resilience of academics as their capacity to learn from and adapt to stress; our definition is perhaps less about individual personality characteristics and more associated with the relational aspect of the socioecological higher education ecosystem. There are, however, limits to resilience and its potential effects on education quality and student learning. Given higher education’s adverse operating environment and the significant contributions of academics to the knowledge economy and graduate quality, understanding and building the resilience of academics to adapt and succeed has never been more critical.

## Introduction

Higher education worldwide is in an era of transformation (OECD, 2003). The COVID-19 pandemic, which brought about the rapid transition to online learning, has accelerated change and intensified stress by closing international borders. The travel restrictions triggered by the COVID-19 pandemic have significantly impacted university finances, resulting in academic redundancies and job losses (Crawford et al., 2020; Cohrssen et al., 2022; De los Reyes et al., 2021, 2022; Mahat et al., 2022a, 2022b; Mercado, 2020; Mok et al., 2020; OCED, 2021; Rapanta et al., 2020). Even before the outbreak of the COVID-19 pandemic, increasingly regulatory approaches to higher education by government, including systemic assessments of research and reviews of education quality (Locke, 2012, 2014; Teichler et al., 2013), resulted in organizational structural reforms. These reforms, in turn, have led to the emergence and increasing dominance of executive leadership models, the transformation of governing bodies into corporate boards, the weakening of academic disciplines and departments by the creation of schools, the concentration of research into research centers, and differentiation of the academic role with the establishment of education- or teaching-focused academics (Krause, 2020; Locke, 2014; Marginson, 2000, 2007; Ross, 2019; Ross et al., 2022). Since then, executive leadership models have shaped higher education institutions into more corporate enterprises, altered the academic workforce, centralized decision-making, and sought efficiencies and cost savings through faculty and curriculum restructuring. This more corporate approach has widened the differences in values between the executive leadership and scholars, created conflict within the university environment, and imposed constraints on academic work (Winter & O’Donohue, 2012). Concern over restrictions on academic work has led to worrisome predictions about the impacts of this more corporate orientation of higher education institutions on knowledge creation, education quality, and academic freedom (French, 2019; Marginson, 2000, 2007; Weatherson, 2018). Indeed, almost two decades ago, well before the COVID-19 pandemic, the consequence of such differences was described as a “destructive standoff… between traditional academic cultures and modernizing corporate cultures” (Marginson, 2000 p. 29). The present-day tensions between executive leadership and academics continue with no end in sight to the standoff.

Given the importance of universities to economies (Valero & Van Reenen, 2019), it is imperative that we have a better understanding of how academics are responding to the changes that have already occurred and that will continue to be a feature of higher education for the foreseeable future. A better understanding of the responses of academics to stress will enable the development of strategies to assist academics in responding to the adversity more positively, especially for early career academics (ECAs) who are just starting out and education-focused academics who are still finding their way in this rapidly changing landscape (Olsen & Sorcinelli, 1992). The concept of resilience may allow academics to better reach their potential and meet the expectations of high-quality contributions to education and discipline-based research. Given the further uncertainty and change looming for higher education, resilience is critical both for academics and higher education if they are to persist and develop solutions to the challenges facing the planet, such as COVID-19 (Mahat et al., 2022a), and reach the educational sustainability development goals (OECD, 2021).

## The key questions

This review is organized to answer two key questions. First, what are the main forms of stress on academics, especially those that derive from education and teaching in the contemporary higher education ecosystem, both pre- and post-COVID-19? We answer this by summarizing the origins and main sources of stress on academics. Second, what are the responses of academics to stress and is resilience a useful concept for understanding the consequences of stress for academic careers? To answer this, we review the literature on the responses of academics to stress, including in the field of teacher education. We then provide a conceptual framework of resilience that extends the Bronfenbrenner socioecological model to describe how higher educational leaders can implement strategies to reduce the magnitude and time needed for academics to recover from the impact of stress so they can learn from and adapt to these strains. Finally, we offer solutions to build resilience of academics because of the potential negative effects of stress on the retention of ECAs and education-focused academics and the flow-on effects to students and education quality. Understanding and building resilience by academic professionals at various levels in the higher education ecosystem is crucial. The strengthening of the resilience and adaptive capacity by academics is likely to have positive impacts on the resilience of students, to improve research and education quality, and to increase trust in the wider democratic practices of higher education.

## Literature review

We will now conduct a critical narrative review, a leading type of literature review (Green et al., 2006). The aim of the literature review was to identify and comprehensively survey the most significant ideas on resilience within and across fields and unite them in a narrative, conceptual synthesis. As is typical of critical narrative reviews, it involved a nonsystematic search (Green et al., 2006) and compilation of main ideas from several areas and disciplines that have established research traditions on stress and resilience, from educational psychology to ecology. A critical narrative review is an ideal form of literature review to combine novel ideas across fields. The key benefits of the approach are the ability to cast a wider scope in the pursuit of novel conceptual synthesis and insights (Baethge et al., 2019). The decision to use this form of review was intentional, as it allowed a summary of the literature in a way that is not explicitly systematic (Baethge et al., 2019; Green et al., 2006). Although critical narrative reviews are not without criticism (Green et al., 2006), they have the major benefit of reduced risk of bias when collating the sum total of evidence on a topic. To identify the relevant literature, we used several databases and search engines, including Google Scholar (Halevi et al., 2017), the Educational Resources Information Center (ERIC), the Institute of Education Sciences and ProQuest Central for education, the Ecology and Society organization for literature on ecological resilience, and the American Psychological Association (Psyc.Net). The search terms included *academic & resilience*, *ecosystem & resilience*, *ecology & resilience*, *stress & resilience*, *adaptive capacity & resilience*, *stress and academics*, *university & resilience*, *teacher resilience*, *resilience of higher education*, and *academic resilience*. These search terms and engines were used to scan the psychological, educational, and ecological resilience literature over the last decade. In Google, the literature returned on the first three search pages for each search was then read. In ERIC and ProQuest, the first 500 papers were scanned for relevance. Additionally, key literature from the 1960s onward on stress and resilience with citation rates of approximately 10,000 from the fields of ecology and psychology were also added to the nonsystematic critical narrative review. Overall, more than 3000 articles were accessed and 168 were read closely and cited in this review. The rationale here was to take a broad perspective and span across disciplines and timelines to enable a transdisciplinary definition and understanding of resilience from the perspectives of psychology, education, and ecology.

### Sources of stress on academics

Even prior to the outbreak of the COVID-19 pandemic, academics across the globe had been indicating escalating workloads and high levels of stress in research and teaching (Winefield & Jarrett, 2001). The origins of academic stress are both proximal and distal. The proximal origins of stress for academics arise from the immersion in a hypercompetitive environment where rejection and criticism are normal features of everyday life. In more recent times, increasing stress on academics has also derived from predatory journals and conference organizers, adding to an already overwhelming email correspondence. The distal origins of stress for academics arise from decisions made by executive leaders and governments, which cascade down through higher education onto the academics themselves. Such distal origins of academic stress include the increasing pressure on academics to teach a greater number and diversity of students, decreasing funding, reduced opportunities for research (disciplinary or education), and increasing and more complex forms of contract cheating and concomitant breaches of academic integrity. As executive leaders seek to save costs and increase efficiencies, academics also experience increasing administration workloads from change management plans that reduce or redeploy professional staff centrally, decreasing casual budgets, and increasing faculty restructuring and curriculum revisions designed to better align with changing and competitive markets for students (Bone & Ross, 2021; Krause, 2020; Whitchurch, 2009; Whitchurch & Gordon, 2013). Academics also experience increasing stress with expectations to rapidly learn and implement new technologies and from digital transformations unrelated to those attributable to COVID-19 (). Moreover, stress arises when the values and beliefs of academics are in conflict with those of executive leaders whose priorities are increasingly geared toward the economic bottom line rather than the academic and higher education mission (Carson et al., 2013; Chan et al., 2020; Day, 2011; Erikson et al., 2020; Winter, 2009; Winter & O’Donohue, 2012; Winefield, 2000; Winefield et al., 2008). Added to these already significant stressors has been the COVID-19 pandemic.

The COVID-19 pandemic has been an acute and intensive stressor. For many academic professionals on the front lines of educational delivery during the COVID-19 pandemic, there were severe consequences for the work-life balance and productivity (Crawford et al., 2020; Mahat et al., 2022a, 2022b; Mercado, 2020; Mok et al., 2020; Peters et al., 2020; Rapanta et al., 2020).

Chronic stress experienced by academics include critiques and rejections of manuscripts, research grant proposals, and promotion applications, criticisms from peers, negative teaching evaluations by students, increasing academic workloads, the widening gap in values between academics and higher education leadership, changes and reform of the academic role and the constant tensions between academics and professional administrators (Chan et al., 2020; Day, 2011; Del Favero & Bray, 2010; Lee et al., 2021; Ross et al., 2022; Whitchurch, 2019; Winter & O’Donohue, 2012).

Rejection of manuscripts and grant applications by peers has a significant impact on academics because their raison d’être is to construct new knowledge and develop the field or subdiscipline; to do this, they need to publish and bring in grant income. Rejection of manuscripts and grants by peers can also be considered as synonymous with rejection from the exclusive social circle of successful academics (Day, 2011). Even when rejections do not occur, peer reviews can damage self-efficacy when the language is harsh and the tone demeaning (Clements, 2020). Clements (2020) states that the peer review process is “… rife with unnecessary, personal comments that merely served as subjective criticisms of the authors’ competencies, … implying that the authors themselves were illogical and unintelligent” (p. 472). The use of personal remarks to describe a research proposal or study can also entrench disadvantages of certain groups (Silbiger & Stubler, 2019) and is unnecessary when more reasonable and constructive criticism can be used.

Another chronic source of stress for academics is student evaluations of their teaching. The origins of student teaching evaluations date back to the 1920s (Marsh, 1980, 1981, 1982, 1984, 1987; Marsh & Bailey, 1993 and reviews within) with the development of the Students’ Evaluation of Educational Quality (SEEQ). While student evaluations vary in name and content by institution and context, the general purpose of student evaluations is to provide academics with needed feedback to assess their teaching effectiveness, understand what has worked and what has not worked, and identify what needs to change. Student evaluations were also created so that administrators could help future learners decide which units to take. Student evaluations were designed to be a reliable and valid multidimensional construct that reflected the complex, multidimensional nature of teaching and provided academics with student feedback on their teaching effectiveness compared to others (Roche & Marsh, 2000). For example, a teacher may be passionate about a topic but not well organized or be able to explain concepts well but need improvement in assessment and feedback. As Roche and Marsh (2000) stated, “helping people to believe in themselves is often considered to be the most important, but also the most challenging, aspect of fostering successful outcomes in many settings” (p. 439). Criticisms and evaluations by colleagues, peers, and students can lead to social rejection, isolation, and hyper vigilance that involves constantly checking for possible threats (Gornall, 2012). Criticism and negative judgments by students, the government and society that consider higher education as not delivering on its expected role are also powerful.

Another source of chronic stress is the increasing academic workload. Reports commissioned by academic unions have found that 90% of academics work more than the allocated hours they are paid for (Strachan et al., 2012; Winefield et al., 2008). Even before the COVID-19 pandemic, excessive workloads were linked to declines in the mental health and well-being of academic professionals. There are no longer peaks and troughs of the workload but rather work is on-going and relentless throughout the year (Morrish, 2019). Even academics who are not engaged in research as a part of their academic role are expected to contribute to scholarship or the governance of the university. Work intensification, where the “amount of work to be done in a fixed time and the time pressure experienced to undertake and complete that work has increased, i.e., how hard and fast an employee is working in any given period” (Fein et al., 2017, p. 361) has also become a feature of academic work (Lee et al., 2021). Furthermore, the type of work performed by academics is increasingly constrained and dependent on university strategic plans. Only those academics with significant research funding are able to maintain the freedom and flexibility to choose work in an area of interest (Chan et al., 2020; Gornall, 2012). Workload models are found in almost all higher education institutions where teaching, student contact hours, and supervision are tallied, but which do not account for the actual time that tasks take to complete, resulting in demotivation (Vardi, 2009). Paradoxically, while the surveillance of academics is increasing (Karlsen, 2013) and *presenteeism* expected, many academics continually perform *unseen* work, reflecting a compulsive form of *hyper professionality* “where they are always working and always electronically connected” (Gornall, 2012 p. 150).

There is an increasingly widening gap in values between academics and the more corporate approach of executive leadership to workload stress. Studies have found that academics share a deep-seated antipathy to the corporatization of universities (Winter & O’Donohue, 2012). Winter and O’Donohue (2012) surveyed over 952 teaching and research academics at levels up to the rank of professor and found that academic values were first and foremost aligned with the view of universities as places of intellectual rigor and with the essential purpose of academic work being scholarship and student learning. Winter and O’Donohue (2012) also found academics were divided into those who *will* and those who *will not* be *managed professionals* (Rhoades, 1998).

Academics are also under pressure due to educational reforms of the academic role (Ross, 2019; Ross et al., 2022). Numerous studies globally provide evidence of the changing nature of both the academic role and higher education more broadly (Bexley et al., 2011; Coates, 2009; James et al., 2013; Locke, 2014; Marini et al., 2019; Teichler et al., 2013). Over the last decade, there has been an increasing trend toward the differentiation of the higher education workforce and academics have been encouraged to target specific activities (Whitchurch, 2019). Stratification is also occurring with the higher education sector, between academics in different types of institutions (e.g., research intensive versus others), mode of employment (e.g., part time and full time, permanent and fixed term), and between disciplinary groupings and between para-academics and academics (Locke, 2014). There has also been an increase in the diversity of the profession, with a growth in the number of academics who have entered higher education from professional practitioner-based disciplines (e.g., law and health). In parallel, there has been a trend toward the emergence of professional staff with specific specialist functions in education, finance, marketing, recruitment, and student services appointed on the basis of external experience in a wide range of sectors (Whitchurch, 2019). Education-focused academics are increasingly a feature of the higher education landscape, including in research intensive universities (Bentley et al., 2013; Coates & Goedgegebuure, 2012; James et al., 2013). Education-focused academics are under pressure because of uncertain career trajectories in higher education and less opportunity to do disciplinary or education research (Ross et al., 2022). Even higher education leaders are unsure and yet use academics as agents of institutional change (Henkel, 2002, 2005) to deliver on societal expectations of graduate employment (Chandler et al., 2002; Deem, 2016; Deem et al., 2008; Diefenbach & Klarner, 2008; Hill, 2012). The evidence of success of these roles which is yet to be determined (Wolf & Jenkins, 2021). 

These changes have not occurred without tensions (Bentley et al., 2013; Dobson, 2000), particularly in the often fractious relationship between academics and professional administrators (Del Favero & Bray, 2010), which has become a major source of stress for academics in higher education. Del Favero and Bray (2010) describe a higher education system with contentious relationships between top-down administrators and academics. Tensions between academics and administrators arise over who has the greatest influence, authority, and right to make decisions and express themselves as a lack of trust (Bone & Ross, 2021; Del Favero & Bray, 2010; Jones, 2012). Growing apprehension and eroded trust between academics and administrators has become a standard feature of higher education institutions (Del Favero & Bray, 2010). The root of this conflict is structural and cultural. Structurally, the increase in the number and type of administrative staff raises concerns that growth in higher education administration has come at the cost of academic positions. Culturally, administrators are seen as cultivating a managerial climate characterized by restructuring and influenced by the external demands of accreditation bodies and graduate demands for employability rather than a focus on academics and disciplines. One reason for this faculty–administrator cultural clash is perhaps that administrators are driven by their collective responsibility to their institutions, whereas academics are motivated by their individual disciplinary-based scholarly pursuits (Del Favero & Bray, 2010). As Larsen et al. (2009) state, there is a need to deal with the “lack of trust between academics and administrators” (p. 14). The widening gap between academics and administrators is important to resolve because it has major implications for academic resilience (Del Favero & Bray, 2010; Larsen et al., 2009). Certainly, there needs to be movement toward a consensual relationship that is transparent, accountable, equitable, inclusive, and built-on trust (Sheng, 2013).

A significant source of acute stress for academics in recent times is the COVID-19 pandemic. As successive waves of COVID-19 infections spread around the world, lockdowns were enforced, international borders were closed, and academics pivoted to working online almost overnight (Chronicle of Higher Education, 2020). The myriad of challenges created for higher education by the COVID-19 pandemic are likely to continue for several years. Some commentators have offered graphic descriptions of the consequences of the COVID-19 pandemic. For example, ) claim that the impact of COVID-19 is similar to “well-known aspects of academics’ recent history” with “professional dysfunction and disturbance, of inequality, exploitation and neglect; of confidence and trust abused and squandered; of disempowerment, displacement, and marginalization; of self-concept on trial and in tatters; of vulnerability and helplessness; and of the loss of a much maligned past superseded by the perceived machinations of digital dystopia and threat of professional oblivion” which has “supercharged a sense of existential panic among academics” (Watermeyer et al., 2021a p. 638). It will be important for future studies to disentangle the actual impacts of universities’ responses to the pandemic from the immediate or distal perceptions of academics to stress related to the disruptions caused by the COVID-19 pandemic experienced in “the heat of the moment.” We should also be careful about the influence of such dystopian representations on the morale of academia. That said, it remains the case that academics’ experience with stress and the impacts of the COVID-19 pandemic have been shared internationally and is multidimensional (McGaughey et al., 2021; Shanker et al., 2021; Table 1).

**Table 1:** Summary of studies which quantified the impacts, both afflictions and affordances or negative and positive, on higher education of COVID-19 in 2020–2021

Afflictions or negative outcomes	Affordances or positive outcomes	
University mission and values		
Corrosive of academic core values, university mission, and perceived value add from university Imposition of disaster managerialism. University leaders exploited crisis for positional gain and disempowered academics. Disingenuity and fair-weather commitment to social justice and inclusivity		Watermeyer et al. (2021a) Watermeyer et al. (2021b)
Over-riding fear, universities, and governments turn the tragedy of the coronavirus and its inescapable economic impacts as a means to legitimately carry through pre-existing plans for cost cutting Legitimize cost cutting		Watermeyer et al. (2021a) McGaughey et al. (2021)
Financial crisis predated COVID-19 would be intensified		McGaughey et al. (2021)
Emptying of university campuses, the abandoning (and necessary repurposing) of halls of residence, student unions, and the like		Watermeyer et al. (2021a)
Intensification of corporate character of universities and more stringent auditing and distribution of finances		Watermeyer et al. (2021b)
Increased academic role specializations (uncoupling of research from teaching) and hardening of anti-intellectualism culture		Watermeyer et al. (2021b)
Increased academic surveillance Intensified top-down governance-weakened trust. Weakened professional autonomy and restricted freedom of choice		Watermeyer et al. (2021b) McGaughey et al. (2021)
	Reassess reliance on international students and embrace domestic students	Shanker et al. (2021)
Work intensification and change		
Massive work intensification, skills cul-de-sac, and erosion of work-life balance. Exacerbating compulsive working and hyper professionality. Digital fatigue Normalized work intensification and sanctioned job cuts		Watermeyer et al., (2021a, 2021b)
Deteriorating work conditions (especially for those whose work intensification might only further augment with depletion of staff numbers), collapse of responsible workload allocation		Watermeyer et al. (2021a)
Intensifying performance evaluation from students Intensified performance demand and evaluation		Watermeyer et al. (2021a) McGaughey et al. (2021)
Technological deficiencies, feelings of ill-prepared and lacking in confidence		Watermeyer et al. (2021a)
Risk of brain-drain and lost generation of academic talent, ECAs unable to find—or disincentivised to find—a first perch on the academic job ladder		
Office hours radically extended		Crick (2021)
Pedagogy and practice		
Rapidly re-design pedagogy and practice, having to learning and assessments, and inability to effectively deliver core computer science topics, such as programming, group projects, and lab-based modules		Crick (2021)
Pedagogical role of academics now purely didactic, transmissional, and therefore regressive		Watermeyer et al. (2021a)
Different pedagogy required for distance teaching and learning and that it is a challenge for faculty to seamlessly make this sudden and unprepared shift from face to face to distance teaching and learning		Marinoni et al. (2020)
	Fuller and sustained utilization of digital pedagogies Online migration step change in the professionalization of academics as teachers far exceeding the tokenism of pedagogic credentialism and other recent efforts to incentivise best or better practice Academics pushed to engage with digital technologies	Shanker et al. (2021) Watermeyer et al. (2021a) McGaughey et al. (2021)
	Renewed focus on the importance of digital skills	Crick (2021)
	Opportunities for pedagogical experimentation Online migration represented far more than just a pedagogical sticking plaster and instead an unparalleled opportunity for pedagogical reinvention	Shanker et al. (2021) Watermeyer et al. (2021a)
	Opportunity to meaningfully co-construct and engage with learners, to give them agency, and to empower them as future digital citizens	Crick (2021)
	Experience of working and teaching from distance as an important opportunity to learn from this exceptional situation and to propose more flexible learning possibilities, explore blended or hybrid learning, and mixing synchronous learning with asynchronous learning. Capacity building of staff and faculty who have learned and tested new tools and systems to enable distance teaching and learning. It is therefore possible that a shift in mindset is happening or that this experience has opened a new horizon of opportunities for teaching and learning	Marinoni et al. (2020)
	Opportunity for innovative assessment	Shanker et al. (2021)
	Reflexive and reflective practice	Shanker et al. (2021)
Research cessation or deprioritization		
Reprioritization of teaching over research, prioritizing of in-person and digital teaching, and neglecting research		Watermeyer et al. (2021b)
Research cessation, emergency online migration represented an inescapable diversion from producing research outputs and consequently a potentially insurmountable barrier to achieving job security Research projects not completed or stopped		Watermeyer et al. (2021a) Marinoni et al. (2020)
Fixed-term contracts dependent on research outputs and ability to attract funding Diversion from research outputs and barrier to achieving job security		Shanker et al. (2021) Watermeyer et al. (2021a)
Impact on research particularly research which requires travel for data collection and laboratories Prohibitions on national and international field work—research which have caused field-based research to cease		Shanker et al. (2021) Watermeyer et al. (2021a)
Frozen, reduced, and canceled research funds		Shanker et al. (2021) McGaughey et al. (2021)
Significant effects for staff seeking career permanence, progression, or promotion		Shanker et al. (2021)
Female academics most disadvantaged in the crisis and research productivity sacrificed to fulfill their role as primary caregiver		Watermeyer et al. (2021b)
	Reprioritization of teaching over research	Watermeyer et al. (2021b)
Job Insecurity and financial loss		
Heightened occupational precarity and insecurity		Watermeyer et al. (2021a)
Non-renewal of fixed-term academic contracts. Elevated precarity for casualized academic staff Damaged ECAs job prospects, lack of career advancement, and increased casualization		Watermeyer et al. (2021b) McGaughey et al. (2021)
Contraction of job security and career opportunities Increased job insecurity and fomenting culture of fear, neutralizing any counteraction or resistance, and emboldening autocratic decision-making Concerns of escalated job precarity		Watermeyer et al., (2021a, 2021b) McGaughey et al. (2021)
Threat of job losses, linked to predictions of lower levels of student recruitment, career stasis, and overall contraction of academic labor market		Watermeyer et al. (2021a)
Labor casualization and hyperexploitation of remaining staff saved from forced redundancy would have more precarious employment		Watermeyer et al. (2021b)
Mental Health and Inequity		
Escalation of work-related stress and waning resilience Work-related stress, work-based harm, boundary between work and home compromised		Watermeyer et al. (2021b) de los Reyes et al. (2021) McGaughey et al. (2021)
Isolation, loneliness, and decrease in mental health and well-being		Shanker et al. (2021) Watermeyer et al. (2021b)
Unequal impact on women with children and caring responsibilities Female academics, typically disproportionately involved in household and pastoral activities, most disadvantaged by the home-based work and online Work-based inequalities		Shanker et al. (2021) Watermeyer et al. (2021a) McGaughey et al. (2021)
Increase in pastoral side of job and significant time in a counseling capacity Student disclosures of health and well-being problems		Watermeyer et al. (2021a) McGaughey et al. (2021)
Long hours, fatigue and exhaustion, and destabilization of work-life balance		McGaughey et al. (2021)
Health and well-being, workload, and job fragility. Worried about mental health and well-being, being always on call. Digital fatigue		Crick (2021)
Concern for students if they become disengaged with online. Worried about their mental health and well-being. Concern about how well we can attend to their pastoral needs		Crick (2021)
	Increased workplace flexibility, less travel, for commuting and conference	McGaughey et al. (2021)
	Digital education opportunity for enhanced social connectivity and inclusivity	Watermeyer et al. (2021a)
	Academic communities closer together, ironically at a time when they are physically most apart	Watermeyer et al. (2021a)
	Trust in younger, less experienced yet ostensibly more technologically adept colleagues	Watermeyer et al. (2021a)

Similar to the responses to other crises, the COVID-19 pandemic has resulted in job losses and cuts to academic staff positions. For those remaining staff, there have been concerns about work intensification, but it is too soon to determine whether this reflects a greater scrutiny of performance and an acceleration of the corporate character of universities (Watermeyer et al., 2021b; Table 1). It seems evident that COVID-19 has caused a reprioritization of teaching over research, which some consider to have returned teaching to its rightful place of importance. Nevertheless, the closure of campuses and restrictions on laboratory investigations and field work have caused much research and practice teaching to cease, especially in the Science, Technology, Engineering, Mathematics, and Medical (STEMM) laboratory-based disciplines (Peters et al., 2020; McGaughey et al., 2021; Shanker et al., 2021; Table 1). There are valid concerns about the flow-on effect of research cessation and the shift from research to teaching on academic permanence and the achievement of faculty tenure, promotion, and progression (Shanker et al., 2021). COVID-19 has also led to significant job losses, disturbance to pedagogical and pastoral roles of academics, and escalation of work-related stress for the academics who remain (Watermeyer et al., 2021a, b). Academics report stress and waning resilience, fatigue and exhaustion, and a destabilization of the work-life balance. Furthermore, these impacts are unequally experienced by women with children and those with caring responsibilities (McGaughey et al., 2021; Shanker et al., 2021).

### Responses of academics to stress

How have academics responded to these stressors? To understand the responses of academics to these sources of stress, we summarize below what is known about the main impacts of these stressors on academics, including any positive adaptive responses to potentially negative stress.

As expected, responses by academics to the stress of rejection and criticism is usually, but not always, negative. A negative response to rejection can be counterproductive and lead to reduced effort and the avoidance of further research. More positive responses to rejection entail thoughts, such as “I can learn from this,” as opposed to “I’m useless,” and may lead to more positive actions, including submitting a manuscript to another journal or moving on to another project, rather than ruminating (Chan et al., 2020). Rejection and criticism can also create a “battle-hardened academic” better able to accept and more immune to rejection and criticism (Chan et al., 2020, p. 457). These “battle-hardened” (Chan et al., 2020, p. 458) and sometimes older and more experienced academics have learned to emotionally detach themselves from rejection and, consequently, are able to leverage negative feedback through criticism to become more productive. Academics, like students, however, vary in their *rejection sensitivity* (Butler et al., 2007). Rejection-sensitive authors, upon receiving a rejection, may engage in more social monitoring, scrutinizing interactions with others to see if they will be rejected, or may avoid discussions of rejections in an attempt to manage others’ impressions of them while cognitively enhancing the value of the journals in which they have published (Pickett et al., 2004). Rejection sensitivity also influences cognition, perception, self-regulation, emotion, motivation, and performance and has been found to result in dysfunctional coping mechanisms (Downey & Feldman, 1996; Frydenberg, 2017; Kaiser & Kaplan, 2006). Rejection sensitivity is a particular concern in academia; because it is a dynamic construct that occurs frequently, the potential for developing rejection sensitivity among academic professionals is high (Day, 2011). Developing such sensitivity is ultimately counterproductive to building resilience. Although some faculty are able to utilize rejection to build resilience, in the worst-case scenario, academics may respond to rejection by abandoning an academic career and higher education (Day, 2011),

Responses of academics to student evaluations are complex. Most simply, in response to poor student evaluations, academics may respond negatively and defensively as they rationalize their poor performance to protect their self-concept (Arthur, 2009; McKeachie, 1979). Roche and Marsh (2000) emphasize the importance “of teachers’ perceptions of their own teaching effectiveness—their teacher self-concepts and the flow-on effect of self-concept on motivation, behavior, and value” (p. 440). Studies have found convergence between the academic self-concept and student evaluations. That is, academics adjust their perceptions upward or downward in response to student evaluations (Marsh & Roche, 1997, 1999, 2000; McKeachie, 1979). Marsh and Roche (2000) found that professors who receive poor ratings can become anxious and defensive and may adopt unhelpful *self-serving* rationalizations in which they attribute the poor rating to external biases, to protect their self-concept. Academics who receive lower than expected ratings by students may respond with denial and defensiveness and may reject student evaluations overall as a valid source of information. In such a situation, academics may direct their attention away from improving their teaching practice and toward alternative activities, such as research and university governance. Even when academics agree with poor student evaluations, they may find themselves at a loss about how to improve (Marsh & Roche, 2000). Roche and Marsh (2000) state, “it is not surprising that many university teachers lack confidence about their teaching effectiveness and may not know how to improve, even if motivated do so” (Marsh & Roche, 2000 p. 446). Similar to rejection sensitivity, the responses of academics to negative feedback vary. Moore and Kuol (2005) found that while academics respond positively to positive feedback, in response to negative feedback, half respond negatively and half react positively. Academics whose response was positive to negative feedback acknowledged that they would change something in their class to address the feedback (Moore & Kuol, 2005). Such positive responses to the stress of negative feedback are similar to learning from the experience of rejection of a manuscript or grant. Given that rejection is here to stay and has a greater impact at the beginning of an academic career, it is especially important that ECAs learn coping mechanisms to normalize rejection and use the negative feedback to improve the quality of their work, avoiding the development of rejection sensitivity (Conn et al., 2016; Day, 2011; Mantai, 2017; Matthews et al., 2014).

In contrast, those academics who respond negatively to negative feedback, even though they may embark on a realistic commitment to improvement, risk dismay, rejection, and withdrawal from a commitment to developing teaching effectiveness (Moore & Kuol, 2005). Rather like the case of the battle-hardened academic (Chan et al., 2020; Day, 2011), negative responses toward negative feedback likely become less frequent with more experience (Arthur, 2009). When negative feedback occurs, adaptive processes need to be put in place to provide the support necessary to identify issues and solutions, especially given that so many faculties are not trained in education or pedagogy. If support is provided, the worst-case scenario is that academics resort to manipulative strategies by lowering standards or awarding students very high grades in response to negative feedback (Marsh & Roche, 2000). Acceptance, rather than rejection, of negative feedback in student evaluations can build resilience.

Unfortunately, the shortcomings of student evaluations have recently received greater attention than their benefits (Fan et al., 2019; Frederike et al., 2017; Hamermesh & Parker, 2003). Studies have found strong biases against females or culturally diverse non-native English speakers (Fan et al., 2019; Frederike et al., 2017; Kaschak, 1978; Sinclair & Kunda, 2000). In some cases, the feedback received by female faculty is 37% lower than that of their male colleagues (Frederike et al., 2017), especially at the upper end where the biases are strongest against young women (Boring, 2017; Frederike et al., 2017). There is also evidence that good-looking (Hamermesh & Parker, 2003) or easy-grading (Greenwald & Gilmore, 1997; Neath, 1996) academics receive more positive student evaluations. While these biases support arguments that student evaluations should not be used for judging performance, tenure, and promotion, regardless of their value (Zabaleta, 2007), they can also get in the way of academics using them effectively as instruments of valuable feedback. Overall, the responses of academics to student evaluations matter because they influence the take up of reflective practice, professional development, and the potential to improve (Arthur, 2009; Moore & Kuol, 2005).

The response of academics to the more corporate higher education enterprise has been either to acquiesce or, conversely, to defend their position, practice, and identity (Winter, 2009). Defensive responses of academics include unionizing and protesting about the constraints on the academic enterprise and real reductions in academic freedom (Becher & Trowler, 2001; Teichert et al., 2013; Weatherson, 2018). Such a defense, however, costs energy and time, which could be more effectively allocated to other activities, and erodes resilience. As Whitchurch and Gordon (2013) found, “the psychological impact of change [in higher education] cannot be underestimated….listening, empathetic skills were seen as vital” (p. 225). To build resilience in academics, executive leadership must understand scholarly pressures and build relationships of trust (Whitchurch & Gordon, 2013). To continue with an autocratic and authoritarian executive management leadership style—including the outsourcing of enterprise bargaining agreements to large multinational professional services firms and “spill and fill” restructuring processes—erodes trust, productivity, and academic resilience.

Systematic assessments of research and underperformance of academics in research have led to the establishment of education-focused roles. The responses of university leadership to educational-focused roles are positive and are viewed as the single most powerful force for reshaping higher education (James et al., 2013; Norton, 2016; Probert, 2013, 2015). Nevertheless, the response of academics to education-focused roles has been mixed (Probert, 2013, 2015; Ross, 2019; Ross et al., 2022; Whitchurch & Gordon, 2010). While some academics view changes to the academic role as an opportunity to focus on teaching rather than research (Bush et al., 2008; Flecknoe et al., 2017; Probert, 2013), others, especially those who have been transferred from traditional teaching and research role to education-focused roles due to underperformance in research, consider it unconscionable (Probert, 2013, 2015; Ross, 2019; Ross et al., 2022). Even academics in executive leadership roles express concerns that the removal of research from the academic role will erode research-led teaching (Schmidt, 2019) and lead to permanent changes to academic identities (Henkel, 2002, 2005). There are reasons for concern, given that transforming the academic role will more likely affect women and entrench their existing underrepresentation in research roles at the senior levels in higher education (Bell, 2009, 2010; Diezmann & Grieshaber, 2019; Ross, 2021).

Positive responses to the COVID-19 pandemic have been far less visible than the negative reactions to the acute stressful experiences. Responses of academics to the stress and adversity of COVID-19 have been described by Watermeyer et al., (2021a; Table 1) as *afflictions* and *affordances* or negative and positive outcomes. The positive responses of academics to the COVID-19 pandemic include the reprioritization of teaching over research and opportunities for novel pedagogical experimentation and ensuing reflective practice (Shanker et al., 2021; Watermeyer et al., 2021a; Table 1). Academics report positive changes from remote working, including increased flexibility and greater social connectivity and inclusivity, which is ironic given that this has been the time when academics have been physically the furthest apart (McGaughey et al.; 2021; Watermeyer et al., 2021a; Table 1). There have been several reports that the COVID-19 pandemic has done more for digital transformation and online learning than any other development in higher education (Dietrich et al., 2020). Opportunities for change in the curriculum and in teaching approaches are being widely discussed (Bryson & Andres, 2020; Dietrich et al., 2020; Gonzalez et al., 2020; Kay et al., 2020; Kedraka & Kaltsidis, 2020; Lyons et al., 2020; Peters et al., 2020; Rapanta et al., 2020). Increased emphasis on pedagogy and uncertainty about what to leave behind and what to carry forward provide hope for a positive outcome (Peters et al., 2020) from the experience of great adversity by scholars (de los Reyes et al., 2021). Despite the pressures from COVID-19, academics at different stages in their careers and in different global contexts have demonstrated sustained engagement (Cohrssen et al., 2022). Studies have suggested that institutions need to support academics systematically and sustainably in times of adversity (Mahat et al., 2022a, 2022b); this will build resilience and help them navigate what is a complex and changing higher education ecosystem (de los Reyes et al., 2022).

So how do we build the conditions that create positive responses to stress and optimize the resilience of academic professionals throughout the higher education ecosystem? We answer this first by defining resilience, reviewing what is known about resilience in teacher education and finally by outlining a framework or model to reduce the impact of negative stress by building strategies for academics to create positive response techniques to stress. This can be particularly useful when higher education faces a crisis, such as the COVID-19 pandemic.

## Resilience of academics

**Fig. 1:**
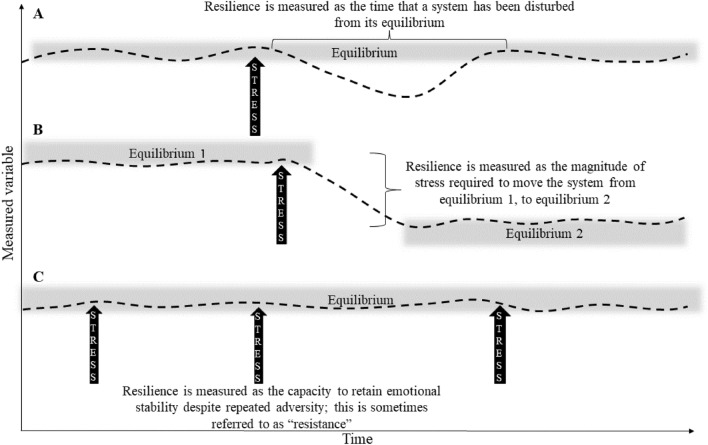
Resilience measured as the **A** time or **B** magnitude of stress that the system or individual academic has been disturbed from equilibrium. **C** Capacity of the system or the individual academic to endure despite repeated adversity or stress

Multiple disciplinary fields over the last half century have explored responses to stress and resilience of complex systems and individuals in the face of adversity (Carpenter et al., 2001; Folke et al., 2004; Frydenberg, 2017; Gu, 2014; Gunderson, 2000; Karlson et al., 2013; Masten, 2001; Walker, 2019). Resilience is broadly defined as the capacity of an ecosystem, society, or individual to *bounce back* and recover from change and stress, whether stress is at a small scale, such as a curriculum or faculty restructuring or a full-blown crisis, such as COVID-19, which has catapulted resilience into the everyday vernacular (Gunderson, 2000; Walker, 2019, 2020). Resilience was first used in the field of engineering to describe systems that resisted stress by not changing (Holling, 1973, 1996). Gunderson, (2000) drawing on the earlier work of Holling (1973), defines resilience as the magnitude or time required for a complex system to return to an equilibrium or steady state following stress, i.e., the duration that the system or individual is pushed away in a negative direction from equilibrium by stress (Fig. 1) or as the time taken to return to an equilibrium (Gunderson, 2000), i.e., when a complex ecosystem or a component of an ecosystem moves from one state to another, the magnitude or time taken for this change to occur is the resilience (Fig. 1).

Psychologists utilize a broader definition of resilience described in terms of the individual rather than the ecosystem (Carver, 1998; Earvolino‐Ramirez, 2007; Masten, 2001; Tugade et al., 2004). Resilience is the emotional response of an individual that entails enduring or bouncing back and overcoming stress. Resilience is also considered the capacity to respond to repeated or cumulative stress and maintain emotional equilibrium, rather than how one copes with a single adverse event (Fig. 1C). Responses to coping with stress include strong social connections and a more positive mindset, which are known to increase resilience and ameliorate stress (Frydenberg, 2014). Although resilience was originally thought of as an extraordinary attribute, more often it is now thought of as a normal and ordinary response to the frequency of stress which is needed to endure and overcome adversity (Masten, 2001; Schoon, 2006).

As the frequency of global disasters such as the COVID-19 pandemic and climate change increases, resilience by ecosystems, society, and individuals has become part of our everyday language. Importantly, multiple fields agree that resilience is not simply about bouncing back (Walker, 2019, 2020), but about having the *adaptive capacity* to *learn* from adversity and stress. The concept of resilience has been used in the fields of ecology, psychology, and more recently school education to conceptualize the capacity of ecosystems, societies, and individuals to have a positive response to stress and, thereby, maintain identity; for example, in the case of a teacher being retained in the school ecosystem. In contrast, resilience has rarely been used to conceptualize how academics respond to change and adversity (but see the recent study by de los Reyes et al., 2021; Mahat et al., 2022a, 2022b and references within).

Overall, remarkably, responses of academics to the multiplicity of stressors in a contemporary university are not always negative, providing evidence of the capacity of academics to *learn from or adapt* to stress and, as a consequence, have the same function and structure, maintain the same identity—i.e., to remain much the same type of system—persist in the face of setbacks, and build resilience. However, resilience has limits, which can be thought of as *tipping points*. Tipping points are reached when the cumulative effect of the stress and challenges related to significant, traumatic events do not build resilience, are counterproductive, and tip over a threshold of tolerance to an alternate state (Gladwell, 2000; Hughes et al., 2003, 2005). Once a threshold is breached, an ecosystem, organization or teacher can tip over to an alternate, undesirable state and, in the case of a teacher or an academic, become lost from the system (Gu, 2014). When an ecosystem, organization, or teacher are close to the limits or threshold of resilience, even a small amount of stress can breach the threshold and tip the system or individual over to an alternate state or individual collapse. These alternate states are difficult or almost impossible to reverse; for example, when the COVID-19 pandemic resulted in the replacement of face-to-face lectures with online lectures. An academic may also tip over a threshold of tolerance in a higher education context, from a small amount of stress that has been building over time to a tipping point, in which the individual leaves the system. Paradoxically, being pushed to the limits and adapting to stress at the boundaries of thresholds can actually build resilience, i.e., avoiding stress does not build resilience. Repeated exposure to stress can also build resilience and act as an inoculation against subsequent stress, rather like a vaccination—also known as *stress inoculation* (Parker et al., 2006; Ross et al., 2022). Resilience should not always be thought of as a good state; there are some undesirable ecosystems where resilience prevails. There are also times when resilience should not be maintained because a more substantial change is needed. A change from a current to a new and different system is known as a transformation (Carpenter et al., 2001; Gunderson, 2000; Walker, 2019). Transformations require leaders who are intentional and who can move the system away from the status quo and out of a state of denial toward alternatives, i.e., a transformation requires actors in the system to stop doing the same things which are not working and move the system toward change (Walker, 2019, 2020).

While academic resilience has received significant attention in terms of a multidimensional construct and having the capacity to recover from setbacks and failures in learning (Martin & Marsh, 2006, 2008), the resilience of scholars to the stresses of everyday academic life (Chan et al., 2020; Lee et al., 2021) and to the more significant pressure of a pandemic has received less attention (de los Reyes et al., 2021; Mahat et al., 2022a, 2022b). Only recently have studies defined the resilience of academics as “the dynamic process and interaction between an academic and their ever-changing environment that uses available internal and external resources to produce positive outcomes in response to different contextual, environmental, and developmental challenges” (de los Reyes et al., 2021, p. 13). This definition emphasizes the relational aspect of resilience, rather than focusing on the individual as in teacher education (Gu & Day, 2007, 2013), and positive outcomes; however, it does not explicitly refer to the key aspect of resilience, that is, the capacity to learn from and adapt to stress.

How to make academics more resilient may be informed by a better understanding of resilience among schoolteachers which has emerged over the last decade in response to the increasing demands on the teaching profession (Ainsworth & Oldfield, 2019; Day & Gu, 2010; Gu & Day, 2007). This research has focused on the importance of teacher retention and teacher resilience for student performance and the conditions needed to build both student and teacher resilience (Gu & Day, 2007, 2013). As in other fields, the resilience of school teachers is defined as the capacity to bounce back when faced with adversity or stress (Day & Gu, 2010; Gu & Day, 2007, 2013). At first, the basis of teacher resilience was thought to be dependent on individual personality traits, such as self-efficacy and self-esteem. Personality differences were used to explain the variation in responses of teachers to adversity and the subsequent reasons for teacher retention or loss (Bonanno, 2004; Luthar & Brown, 2007). However, once the relational aspect was recognized, teacher resilience was viewed as dependent on the level of trust and support among colleagues and principals in the social and organizational structure of a school. Such a relational view of resilience, rather than as an individual personality trait, puts more responsibility on school governance to create a supportive environment (Beltman et al., 2011; Day, 2011; Day & Gu, 2010; Gu, 2014; Gu & Day, 2007; Luthar & Brown, 2007; Mansfield et al., 2016; Ungar et al., 2012; Ungar et al., 2013). Ungar (2012) emphasized the difficulty in reconciling the relational aspects of teacher resilience independent of individual personality traits, such as self-efficacy and self-esteem, which also depend on good relationships (Ungar, 2012). It is now thought that teacher resilience, that is, the capacity of teachers to bounce back from adversity, reflects the interaction between individual personality resources such as self-efficacy and self-esteem and the relational aspects of the internal and external social and organizational environments (Beltman et al., 2015; Gu, 2014; Ungar, 2012). When these interactions are positive, they build resilience and form the basis of teacher well-being and job satisfaction as well as student performance; when they are less than positive, they erode resilience and lead to teacher burnout and departure from the profession (Beltman et al., 2011). Ungar et al.’s (2013) model can be applied to an understanding of academic resilience because academics have the capacity to draw on resources available to them to build resilience, including interrelationships, support from colleagues, and the executive leadership; at the same time, however, these colleagues are also their direct competitors. Academic professionals within higher education are individualistic and operate in increasingly competitive environments, where achieving individual goals aids institutional performance. In contrast to teachers in schools, academics in universities are judged on their performance primarily in research and grant winning rather than in education and teaching quality. Unlike teachers, scholars have allegiances to cultures of disciplines rather than institutions (Becher & Trowler, 2001).

Understanding the resilience of academics may also come from better understanding of resilience of the entire higher education ecosystem. Executive leaders can limit the stress on academics by building resilience of the higher education ecosystem through effective functioning and governance (Karlsen, 2013 p. 18). Institutional resilience is the intrinsic ability of an institution to adjust its functions prior to, during, and following unexpected change or stress (Karlsen, 2013) and to develop a highly tuned sense of future developments (Valikangas & Romme, 2012; Wildavsky, 1991). Valikangas and Romme (2012) describe three strategic management practices for institutions to build resilience. These are cultivating foresight, rehearsing non-routine behaviors, and building an experiment-orientated community. They also suggest that resilience of organizations has two dimensions: operational resilience, i.e., the ability to bounce back after a crisis, and strategic resilience, which is the ability to turn a crisis into an opportunity (Valikangas & Romme, 2012). Since the mid-1980s, attempts to create more adaptive governance structures have been a priority of higher education (Larsen et al., 2009; Whitchurch & Gordon, 2013). Building future resilience in higher education requires the resolution of conflicts and dilemmas between executive leadership, administrators, and academics to build trust through adaptive management practices which cultivate foresight and experimentation both at an operational and strategic level; thus, when a crisis occurs, such as the COVID-19 pandemic, it can be effectively weathered. Such resolutions are needed for arguments over parallel academic and administration decision-making structures, the mix and influence of representation of internal and external stakeholders, including domestic and international students, the balance between centralization and decentralization, and the redistribution of authority within institutions (Larsen et al., 2009).

## A framework to build the resilience of academics

**Fig. 2:**
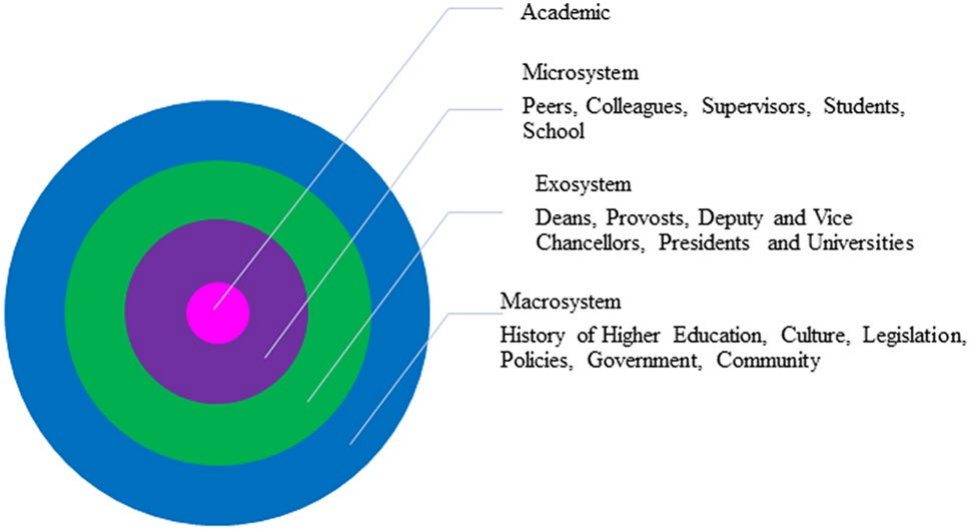
Bronfenbrenner’s socioecological model representing the layers of influence and stress on academics in higher education

The resilience of academics can be considered in the framework of Bronfenbrenner’s (1977) socioecological model of development (Ungar, 2012; Ungar et al., 2013). Bronfenbrenner (1977) conceived of a child’s environment as a series of nested babushka dolls and defined human development “as the person’s evolving conception of the ecological environment and his [sic] relation to it, as well as the person’s growing capacity to discover, sustain, or alter its properties” (p. 9). Bronfenbrenner’s (1977) model positions the child in a set of nested relationships in his or her environment, where the individual is influenced by the environment and, in turn, the environment influences the individual (Bronfenbrenner & Ceci, 1994; Ungar, 2012; Ungar et al., 2013), shown as a series of concentric circles (Fig. 2). At the center of the circle is the child or individual, and the circles that then span out from the individual are the microsystem, the mesosystem, the exosystem, the macrosystem, and the chronosystem (Bronfenbrenner, 1977; Bronfenbrenner & Ceci, 1994; Guy-Evans, 2020). The first circle is the microsystem, where the interactions between the individual and the environment are bidirectional; each can influence and change the opinion of the other. The mesosystem is where the interactions among microsystems take place. The exosystem is the environment that does not directly contain the individual but has significant influence. The macrosystem is the influential culture in which an individual is immersed and which influences the individual’s beliefs. Finally, the chronosystem is the place of events that influences individuals and occurs over a lifetime (Guy-Evans, 2020; Fig. 2). Ungar et al. (2013) used Bronfenbrenner’s model to conceptualize an ecological model of resilience that can also be applied to academics in the higher education ecosystem. An academic is in the microsystem with direct influence from peers, colleagues, students, and supervisors. The exosystem is the environment that does not directly contain the individual, but exercises significant influence over that individual, e.g., decisions made by deans and vice chancellors. The macrosystem is the influential culture in which an academic is immersed, which influences beliefs, i.e., the culture of higher education. Ungar et al. (2013) state, “this way of conceptualizing resilience means that individuals are not always the most important locus for change” (p. 357). Resilience in academics, similar to resilience in school teachers’ experiences of stress, is not all explained by the individual characteristics of an academic but, rather, is a product of the multiple systems in which the academic interacts and is influenced by the relationships in the exosystem and mesosystem (Beltman et al., 2015; Mansfield et al., 2016; Ungar et al., 2013). As Ungar et al. (2013) states, “our understanding of resilience is shifting in much the same way that Bronfenbrenner shifted the focus on human development from the individual to the multiple systems with which the individual interacts” (p. 349). More recently, Kinchin (2022a, 2022b) has reinforced the conceptualization of a university and individuals within as a socioecological ecosystem. He uses an ecological lens on the professional development of academics and applies Holling’s (1996) adaptive cycles to describe the constructive and destructive processes which occur at different levels (i.e., at the individual, the discipline, and the institution level) and time scales within the university ecosystem (2022b; Kinchin, 2022a).

**Fig. 3:**
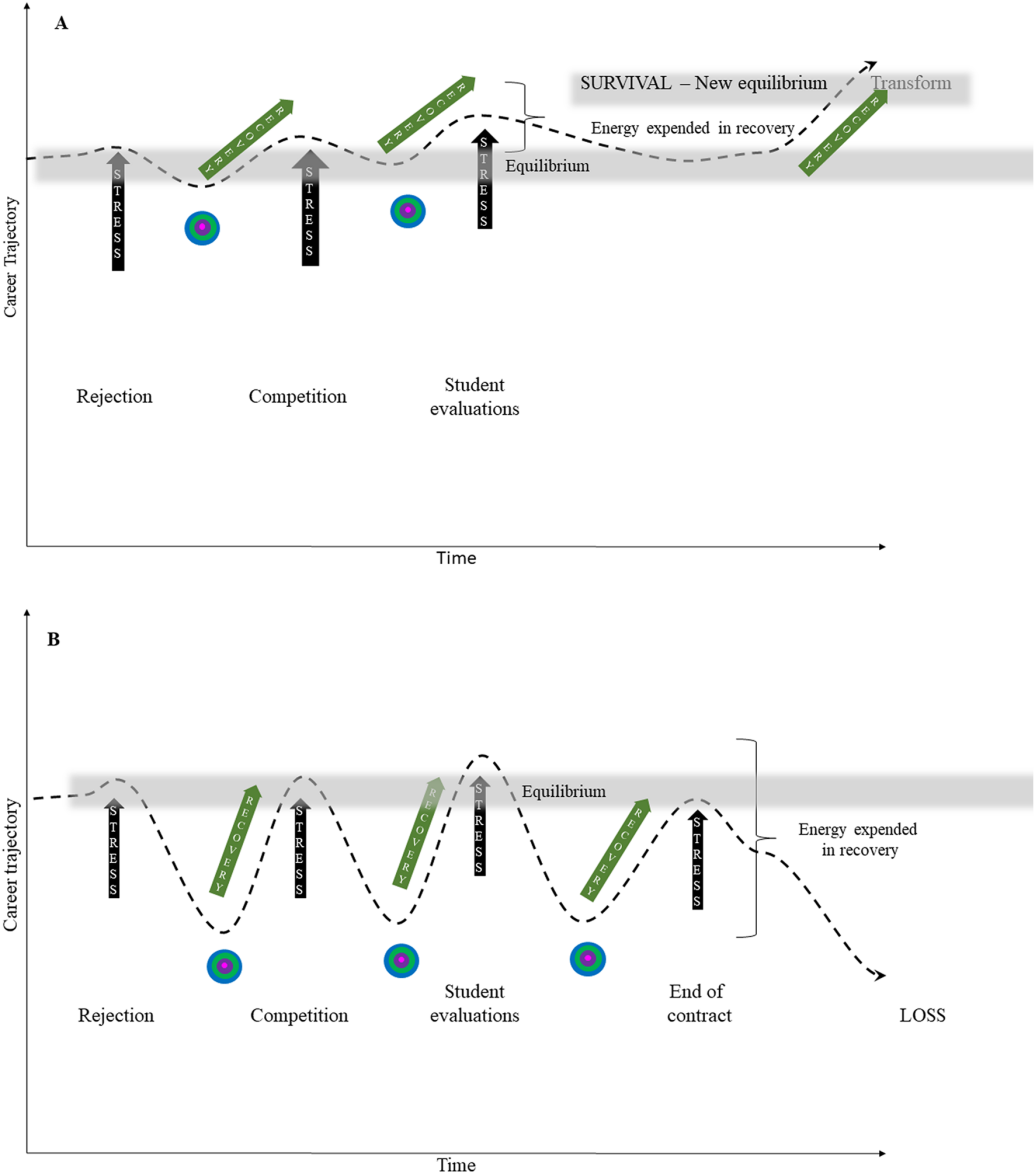
Career trajectory of academic **A** and **B** with stress and recovery periods caused by rejection, competition, and end of contract or loss of tenure. Academic **A** experiences more significant impact to the same stressors than academic **B** and more energy is expended to recover and is lost from the system, whereas academic **B** survives in a new equilibrium. (Black bars are stress and green bars recovery). Bronfenbrenner’s model of human development being present in the recovery stage of academic A. Bronfenbrenner’s model in operation at moments of stress in **A** and **B**, but more effective in **A**

The resilience of an academic is thought to be greatest when there is a moderate period of time between stress-inducing events. In other words, too much stress can be overwhelming, yet not enough stress can result in complacency. Resilience may also be reduced when there are long gaps or short gaps of time between stressful episodes, i.e., long periods without stress and short intervals between stressful events are likely to be equally damaging when a future stress-inducing event occurs (Hughes et al., 2021). For example, consider two career trajectories of academics A and B, with stress and recovery periods caused by rejection, competition, and end of contract or loss of tenure (Fig. 3). Academic individual A experiences less significant impact to the same stressors than individual B and less energy is expended to recover and survive to reach a new equilibrium; conversely, academic B suffers significant impact and becomes lost from higher education. The Bronfenbrenner’s socioecological model can also be integrated and conceptualized to understand the recovery from stress of academic individuals A and B (Fig. 3). Academic A goes on to experience more positive bidirectional relationships in the microsystem and exosystem, while academic B does not. As a consequence academic A survives and may go on to reach a new equilibrium, such as a leadership position or promotion; conversely, academic B does not receive the support expected and needs to find a new career. Transformation applies to both academic A and B: academic A may have transformed and created a new identity as a teacher or researcher, whereas academic B may have found him or herself unable to connect with social networks and mentoring or be open to new ideas led by the leaders in the macrosystem. In this way, the relationships described by Bronfenbrenner’s model are activated at each stress point and can increase the effectiveness of the recovery (Fig. 3). Bronfenbrenner’s socioecological model of resilience moves our understanding of resilience away from the individual, in this case the academic toward a focus on the socioecological factors or the environment that affect academics in the higher education ecosystem, facilitating well-being under stress (Ungar et al., 2013). It provides a useful model for conceptualizing the various stressors on academics and how they are moderated or magnified.

The literature also distinguishes resistance from resilience. Resistance has been considered a complimentary attribute of resilience (Carpenter et al., 2001), and sometimes resistance is distinguished from resilience (Connell et al., 2016). Some systems are capable of absorbing high levels of external pressure without experiencing a measurable disturbance and are, therefore, considered resistant (Fig. 1C). For example, an academic, particularly a battle-hardened academic, may appear resistant to the stress of grant rejection. Nevertheless, the memory of this experience remains and may affect his or her propensity to reapply and a future response to rejection.

## Recommendations and conclusion

Multiple authors have offered solutions or recommendations to the challenges of building resilience, including mentoring, establishing supportive networks of colleagues, and creating institutional cultures that are not hostile but rather collegial. These solutions provide support, soften the impact of rejection and criticism (Chan et al., 2020; Conn et al., 2016; Day, 2011; Hollywood et al., 2020; Mahat et al., 2022a, 2022b), facilitate adaptive capacity, and build success and resilience; and all have become importance because of COVID-19 (Mahat et al., 2022a, 2022b). Such strategies have been implemented in institutions to limit the magnitude of the impact of the stress on academics, particularly those at the early- or mid-career stages in an attempt to socialize stress and decrease the time required for recovery.

In answering the key questions posed in this review, we argue that if academics and higher education leaders can understand and build the resilience of academics, they then are more likely to retain and strengthen performance, particularly in the case of scholars who are at the start of their career, such as ECAs and education-focused academics, who are still learning the system and are vulnerable (de los Reyes et al., 2022; Ross et al., 2022)  The improved resilience of academics will also likely affect students’ performance and education quality (Gu, 2014) as well as contribute to the building of trust in the wider democratic practices in higher education ecosystems. The advantages of conceptualizing academic resilience as relational as described by Bronfenbrenner are, first, that it provides both academics and higher education leadership with a framework to deliver and build a culture that is more trusting than that which currently exists and, second, it provides a powerful heuristic for academics and higher education leaders to navigate what is clearly a changing and uncertain landscape. The problem with the widening gap in values between academics and higher education leadership is that the relationships can become frayed, cascading the negative impacts on academics and then onto students. The socioecological model of Bronfenbrenner provides a framework for conceptualizing influence and finding solutions. Given the future adverse operating environment for higher education, the changing nature of the academic role, the on-going structural reforms, and the uncertainty of the COVID-19 pandemic, understanding how academics and higher education ecosystems learn and adapt to stress and build resilience has never been more urgent.
